# Heterometallic Europium(III)–Lutetium(III) Terephthalates as Bright Luminescent Antenna MOFs

**DOI:** 10.3390/molecules27185763

**Published:** 2022-09-06

**Authors:** Viktor G. Nosov, Arkady S. Kupryakov, Ilya E. Kolesnikov, Aleksandra A. Vidyakina, Ilya I. Tumkin, Stefaniia S. Kolesnik, Mikhail N. Ryazantsev, Nikita A. Bogachev, Mikhail Yu. Skripkin, Andrey S. Mereshchenko

**Affiliations:** 1 Saint-Petersburg State University, 7/9 Universitetskaya emb., 199034 St. Petersburg, Russia; 2 Nanotechnology Research and Education Centre RAS, Saint Petersburg Academic University, ul. Khlopina 8/3, 194021 St. Petersburg, Russia

**Keywords:** metal–organic framework, luminescence, rare earth, europium, lutetium, phase transition

## Abstract

A new series of luminescent heterometallic europium(III)–lutetium(III) terephthalate metal–organic frameworks, namely (Eu_x_Lu_1−x_)_2_bdc_3_·nH_2_O, was synthesized using a direct reaction in a water solution. At the Eu^3+^ concentration of 1–40 at %, the MOFs were formed as a binary mixture of the (Eu_x_Lu_1−x_)_2_bdc_3_ and (Eu_x_Lu_1−x_)_2_bdc_3_·4H_2_O crystalline phases, where the Ln_2_bdc_3_·4H_2_O crystalline phase was enriched by europium(III) ions. At an Eu^3+^ concentration of more than 40 at %, only one crystalline phase was formed: (Eu_x_Lu_1−x_)_2_bdc_3_·4H_2_O. All MOFs containing Eu^3+^ exhibited sensitization of bright Eu^3+^-centered luminescence upon the 280 nm excitation into a ^1^ππ* excited state of the terephthalate ion. The fine structure of the emission spectra of Eu^3+ 5^D_0_-^7^F_J_ (J = 0–4) significantly depended on the Eu^3+^ concentration. The luminescence quantum yield of Eu^3+^ was significantly larger for Eu-Lu terephthalates containing a low concentration of Eu^3+^ due to the absence of Eu-Eu energy migration and the presence of the Ln_2_bdc_3_ crystalline phase with a significantly smaller nonradiative decay rate compared to the Ln_2_bdc_3_·4H_2_O.

## 1. Introduction

In recent decades, rare-earth-element metal–organic frameworks (REE-MOFs) were actively designed and synthetized due to their unique luminescence properties. They are unique platforms for fabricating advanced luminescent materials, which are widely used in various fields of science and technology [1,2,3,4]. The position of the lanthanide ionic luminescence bands strongly depends only on the lanthanide ion, which allows the construction of REE-MOFs with the desired optical properties [5]. Taking this fact into account and considering the high stability, low solubility and toxicity, and highly effective charge transport of Ln-MOFs, they are prospective materials for OLEDs [6,7], luminescent thermometers [8,9], and imaging [10,11,12,13]. Variations in organic linkers in MOFs allow synthetic chemists to form structures with different porosities large surface areas, and high structural flexibility [14,15,16], which allows the use of REE-MOFs as highly selective sensors on organic and inorganic materials [17,18,19,20,21,22,23,24,25]. Typical linkers in REE-MOFs are organic carboxylates due to the simple synthesis of REE-MOFs in undemanding conditions and the unlimited possibilities in MOF design [26,27].

Lanthanide ions possess characteristic luminescence; however, direct UV excitation of them is inefficient because they have very small light absorption coefficients: 4f-4f transitions are forbidden by selection rules. This issue can be resolved using the energy transfer from the excited linker to the lanthanide ion (antenna effect) [28,29]. Aromatic organic molecules such as 1,4-benzenedicarboxylate (bdc) are widely used as antenna linkers due to their effective UV absorbance and pronounced antenna effect [30,31]. Usually, the energy transfer takes place from the lower-level triplet electronic state (T_1_) of the linker molecule but not from the lowest excited singlet state (S_1_). In some cases, the heavy lanthanides increase the rate of S_1_-T_1_ intersystem crossing [32,33,34,35]. The high concentration of luminescent lanthanide ion in homometallic REE-MOFs could result in concentration quenching through Ln-Ln energy migration and, therefore, the drop in luminescence quantum yield (PLQY) [36]. Utochnikova et al. proposed a solution to this problem by doping of a luminescent europium(III) terephthalate with nonluminescent Gd^3+^ ions, which not only diluted the luminescent Tb^3+^ ions, but also increased the probability of intersystem crossing, which increased the PLQY. It was found that Eu-Gd and Eu-Y heterometallic terephthalates were formed in the same crystalline phases. In the current work, we studied a series of luminescent heterometallic europium(III)–lutetium(III) terephthalates MOFs and observed that the substitution of a large amount of Eu(III) for Lu(III) resulted in a crystalline phase change as well as a significant rise in the PLQY.

## 2. Results and Discussion

### 2.1. XRD Results and Analysis

**Figure 1:**
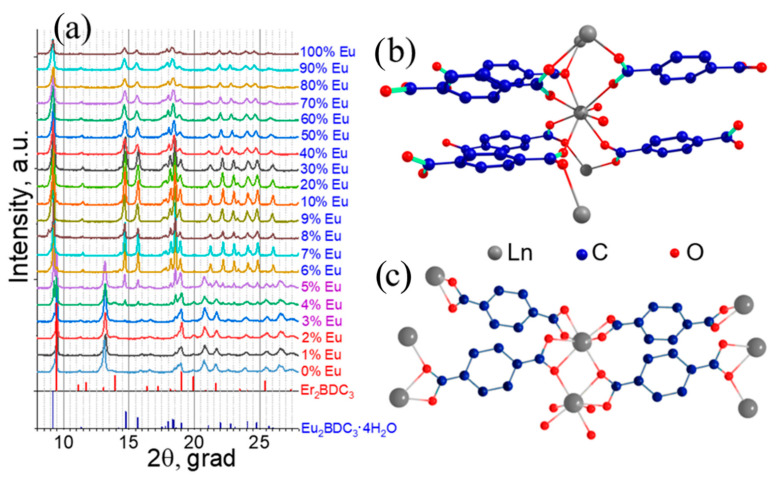
(**a**) The XRD patterns of (Eu_x_Lu_1−x_)_2_bdc_3_·nH_2_O in heterometallic europium(III)–lutetium(III) terephthalate powders from 0% Eu^3+^ to 100% Eu^3+^) and the simulated XRD pattern of Er_2_bdc_3_ and Eu_2_bdc_3_·4H_2_O single-crystals structure taken from refs. [37,38]. (**b**,**c**) The generated crystal structures of Eu_2_bdc_3_·4H_2_O and Er_2_bdc_3_, respectively.

The X-ray powder diffraction (XRD) patterns (Figure 1a) were measured for the range of heterometallic europium(III)–lutetium(III) terephthalates (Eu_x_Lu_1−x_)_2_bdc_3_·nH_2_O; bdc = 1,4-benzenedicarboxylate) with a Eu^3+^ concentration from 0 to 100 at %. An analysis of the XRD patterns demonstrated that in a range of Eu^3+^ concentration of 6 to 100 at %, the samples were isostructural to the Ln_2_bdc_3_·4H_2_O (Ln = Ce-Yb) [37]. This structure, which is common for the rare-earth terephthalates from Ce to Yb [38], was a three-dimensional metal–organic framework (MOF) in which octacoordinated lanthanide ions were bound to the two water molecules and six terephthalate ions through the oxygen atoms (Figure 1b). The analysis of XRD patterns of Eu-Lu terephthalates with 0–2 at % of Eu^3+^ showed that the samples were isostructural to Er_2_bdc_3_ [38], which is a 3D MOF in which heptacoordinated lanthanide ions are bound to the seven oxygen atoms from the terephthalate ions (Figure 1c). At a Eu^3+^ concentration in the range of 3–5 at %, both the Ln_2_bdc_3_·4H_2_O and Ln_2_bdc_3_ crystalline phases were observed. The XRD peaks for heterometallic europium(III)–lutetium(III) terephthalates in Eu^3+^ concentration ranged from 6 to 100 at % and were slightly shifted relative to the XRD peaks measured for Ln_2_bdc_3_·4H_2_O reported previously [37]. To compare these Ln_2_bdc_3_·4H_2_O structures, the refinement of unit cell parameters was performed for some samples with a Eu^3+^ concentration between 6 and 100 at % (Table 1) using UnitCell software [39], which retrieved unit cell parameters from diffraction data using a method of least squares from the 2Θ data of the XRD patterns. Calculation errors also are shown in Table 1. We observed that in the range, the unit cell parameters increased. The observed growth of the unit cell parameters of heterometallic europium(III)–lutetium(III) terephthalates was explained by the smaller ionic radius of the octacoordinated Lu^3+^ (0.977 Å) compared with the ionic radius of the Eu^3+^ ion (1.066 Å) [40]. 

### 2.2. Thermogravimetric Analysis (TGA)

**Figure 2:**
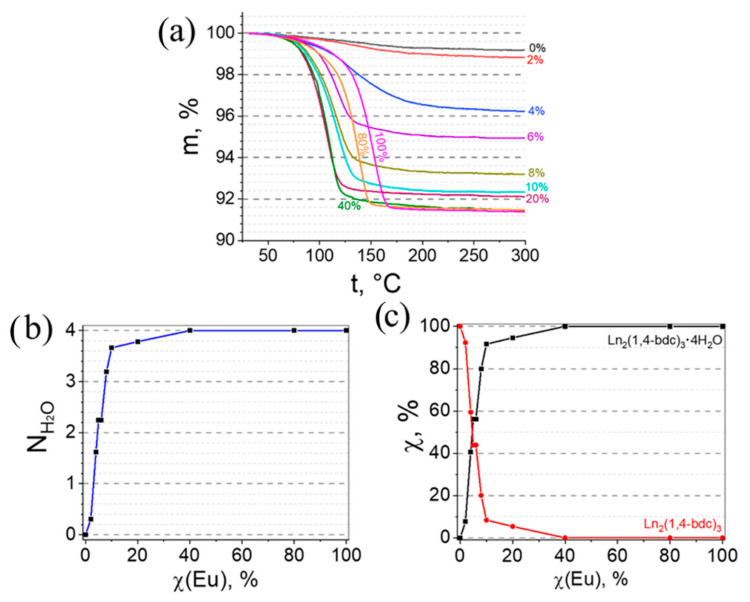
(**a**) Thermogravimetric analysis (TGA) curves showing the mass loss profile of (Eu_x_Lu_1−x_)_2_bdc_3_·xH_2_O during thermal decomposition; (**b**) the number of water molecules per one formula unit in (Eu_x_Lu_1−x_)_2_bdc_3_·xH_2_O; (**c**) the molar fraction of Ln_2_bdc_3_ and Ln_2_bdc_3_·4H_2_O in heterometallic europium(III)–lutetium(III) terephthalates as a function of Eu concentration.

The thermogravimetric analysis (TGA) was carried out for the selected heterometallic europium(III)–lutetium(III) terephthalates in a temperature range of 25–300 °C (Figure 2a). The mass loss was observed at 120–180 °C for all measured samples. As previously reported [38], the mass loss in this temperature range can be assigned to the dehydration of the compounds resulting in the formation of Ln_2_bdc_3_. An analysis of the TGA curves allowed us to calculate the average numbers of water molecules in the heterometallic europium(III)–lutetium(III) terephthalates ((Eu_x_Lu_1−x_)_2_bdc_3_·nH_2_O). We observed that this number increased with the increase in the Eu^3+^ concentration (Figure 2b). An analysis of the XRD patterns demonstrated the presence of two crystalline phases: Ln_2_bdc_3_·4H_2_O and Ln_2_bdc_3_. Therefore, we could estimate the molar fraction of each coexisting crystalline phase (Figure 2c). The molar fraction of Ln_2_bdc_3_·4H_2_O increased along with the Eu^3+^ concentration in a range between 0 and 40 at %. In the Eu^3+^ concentration range of 40–100%, only Ln_2_bdc_3_·4H_2_O was present.

### 2.3. Luminescent Properties

**Figure 3:**
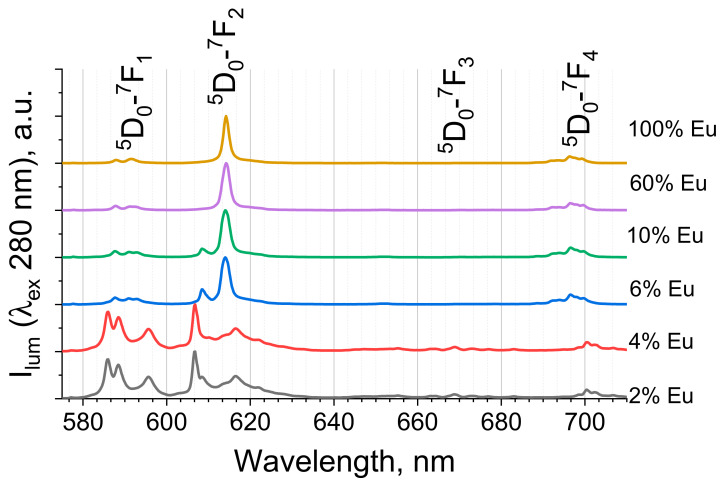
The normalized emission spectra of (Eu_x_Lu_1−x_)_2_bdc_3_·nH_2_O at selected Eu^3+^ concentrations (given in legend) upon 280 nm excitation.

**Figure 4:**
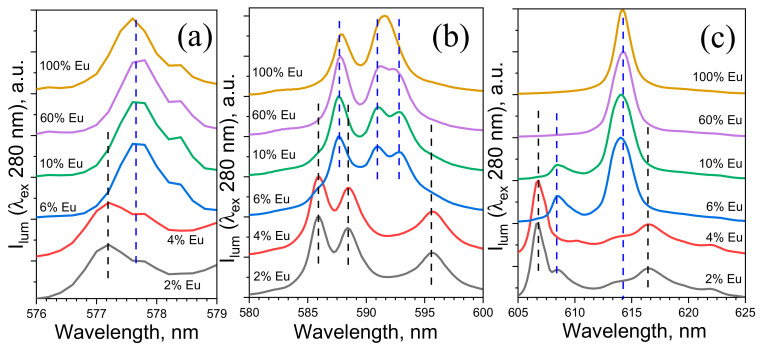
Fine structure of lines in emission spectra of heterometallic europium(III)–lutetium(III) terephthalates normalized at maximum point for (**a**) ^5^D_0_-^7^F_0_, (**b**) ^5^D_0_-^7^F_1_, and (**c**) ^5^D_0_-^7^F_2_ transitions.

The terephthalate ion is a typical linker used in luminescent antenna MOFs [41] due to its intensive UV absorbance [42] followed by efficient energy transfer to the luminescent lanthanide ion. The excitation of (Eu_x_Lu_1−x_)_2_bdc_3_·nH_2_O (λ_ex._ = 280 nm) resulted in emission in the visible range corresponding to ^5^D_0_-^7^F_J_ (J = 0–5) transitions of the Eu^3+^ ion [5] (Figure 3 and Figure 4). Upon UV excitation, the terephthalate ion was promoted into the S_n_(^1^ππ*) state followed by the fast internal conversion to S_1_(^1^ππ*). Due to the heavy atom effect, the S_1_ state efficiently moved to the T_1_(^3^ππ*) triplet electronic excited state [34] via intersystem crossing. The T_1_ state of the terephthalate ion [34] (≈20,000 cm^−1^) had a higher energy than the ^5^D_1_ energy level of the Eu^3+^ ion [5] (≈19,000 cm^−1^) and a significantly lower energy than that of the lower excited state of the Lu^3+^ ion [43] (80,000 cm^−1^). Therefore, an efficient energy transfer from the T_1_ triplet electronic excited state of the terephthalate ion to the ^5^D_1_ energy level of the Eu^3+^ ion occurred. The ^5^D_1_ level of the Eu^3+^ ion then underwent an internal conversion into the ^5^D_0_ energy level followed by the emission into the ^7^F_J_ (J = 0–4) lower-lying levels. 

We observed that the fine structure of the Eu^3+^ emission spectra significantly depended on the Eu^3+^ concentration in the (Eu_x_Lu_1−x_)_2_bdc_3_·nH_2_O (Figure 4). At Eu^3+^ ion concentrations of more than 6 at %, in which the Ln_2_bdc_3_·4H_2_O phase dominated, the emission spectra were similar to that of Eu_2_bdc_3_·4H_2_O [31] and consisted of narrow bands corresponding to ^5^D_0_-^7^F_J_ (J = 0–4) transitions of Eu^3+^: ^5^D_0_-^7^F_0_ (577.6 nm), ^5^D_0_-^7^F_1_ (587.9 and 591.5 nm), ^5^D_0_-^7^F_2_ (614.0 nm), ^5^D_0_-^7^F_3_ (649.0 nm), and ^5^D_0_-^7^F_4_ (697.0 nm) (Figure 3). At low Eu^3+^ concentrations (2 and 4 at % Eu^3+^), in which the Ln_2_bdc_3_ phase dominated, the fine structure of the emission spectra was significantly different. The emission spectra contained ^5^D_0_-^7^F_0_ (577.2 nm and 577.6 nm), ^5^D_0_-^7^F_1_ (585.9, 588.4, and 595.6 nm), ^5^D_0_-^7^F_2_ (606.6, 610.2, 616.6, 619.4 (shoulder), and 621.8 nm), ^5^D_0_-^7^F_3_ (649.0 nm), and ^5^D_0_-^7^F_4_ (700.0 nm) Eu^3+^ narrow emission bands.

^5^D_0_-^7^F_0_ transition is strictly forbidden by the Judd–Ofelt theory; one can observe this transition only for europium(III) ions in coordination sites with C_n_, C_nv_, and C_s_ symmetry. For all measured samples, ^5^D_0_-^7^F_0_ transitions were observed. The analysis of the fine structure of this transition allowed us to determine the number of Eu^3+^ coordination sites because the ^7^F_0_ level was not degenerate and did not split in the crystal field, hence ^5^D_0_-^7^F_0_ could present in the emission spectrum as a single line for one type of Eu^3+^ coordination. The fine structure of the (Eu_x_Lu_1−x_)_2_bdc_3_·nH_2_O emission bands ^5^D_0_-^7^F_0_ is shown in Figure 4a. In the emission spectra of Eu-Lu terephthalates with a Eu^3+^ concentration of 6–100 at %, a single line was observed in the 570–585 nm range (^5^D_0_-^7^F_0_) with a maximum at 577.6 nm, which indicated that Eu^3+^ ions existed in the single-crystal-phase isostructural Ln_2_bdc_3_·4H_2_O. Meanwhile, by using a TGA, we estimated the molar fractions of Ln_2_bdc_3_·4H_2_O and Ln_2_bdc_3_ as equal to 60 and 40%, respectively. Therefore, the single ^5^D_0_-^7^F_0_ emission band (6–100 at % Eu^3+^) can be explained by uneven ion distribution between the two phases: the Ln_2_bdc_3_·4H_2_O crystalline phase was enriched by Eu^3+^ ions. In the Eu-Lu terephthalates with 2–4 at % Eu^3+^, two emission bands corresponding to the ^5^D_0_-^7^F_0_ transition were observed to peak at 577.2 and 577.6 nm, indicating the two different coordination sites of the Eu^3+^ ion. Therefore, in the terephthalates containing 2–4 at % Eu^3+^, europium(III) ions were distributed between two phases, namely Ln_2_bdc_3_·4H_2_O and Ln_2_bdc_3_, which was consistent with the TGA and XRD data. 

The fine structure of the ^5^D_0_-^7^F_J_ emission bands and their relative intensities were very sensitive to the Eu^3+^ ions’ local symmetry. The degeneracy of each spin–orbit level was 2J+1 [5]. Hence, the maximum amount of crystal-field transitions of the ^5^D_0_-^7^F_1_ and ^5^D_0_-^7^F_2_ transitions were 3 and 5, respectively. According to previous studies, lanthanide(III) ions had pseudo-C_4_ symmetry in Ln_2_bdc_3_·4H_2_O (Ln = Tb, Eu) [38]. For Eu_2_bdc_3_·4H_2_O (100 at % Eu^3+^), the ^5^D_0_-^7^F_1_ transition split into two crystal field transitions (587.9 and 591.6 nm), and the ^5^D_0_-^7^F_2_ transition was presented in the emission spectrum as a single line (614.0 nm). Interestingly, for the Eu-Lu terephthalates containing 6–60 at % Eu^3+^, different splitting patterns of the ^5^D_0_-^7^F_J_ transitions in the crystal field were observed. The ^5^D_0_-^7^F_1_ and ^5^D_0_-^7^F_2_ transition split into three (587.6, 591.0, and 592.8 nm) and two components (608.5 and 614.0 nm), respectively (Figure 4b,c). The difference between the abovementioned emission spectra can be explained by the distortion of the coordination polyhedron due to the appearance of structural defects caused by the addition of Lu^3+^ ions, which have a lower ionic radius than europium(III) ions (lanthanide contraction). This resulted in the lowering of the local symmetry of the Eu^3+^ ion and the larger number of crystal-field transitions of Eu-Lu terephthalates compared to the Eu_2_bdc_3_·4H_2_O. The number of ^5^D_0_-^7^F_J_ crystal-field transitions indicated that the Eu^3+^ had symmetry of C_2_ or lower [44]. 

In the emission spectra of Ln_2_bdc_3_ (2–4 at % Eu^3+^), the ^5^D_0_-^7^F_1_ and ^5^D_0_-^7^F_2_ transitions split into three (585.9, 588.4, and 595.6 nm) and five components (606.6, 610.2, 616.6, 619.4 (shoulder), and 621.8 nm), respectively. In the emission spectra of the Lu-Eu terephthalates containing 2–4 at % Eu^3+^, we observed the presence of (Eu_x_Lu_1−x_)_2_bdc_3_·4H_2_O emission bands (weak 608.5 and 614.0 nm signals) because the Eu^3+^ ion was distributed between the Ln_2_bdc_3_ and Ln_2_bdc_3_·4H_2_O crystalline phases. A careful analysis of the Ln_2_bdc_3_ crystalline structure (Figure 1c) allowed us to conclude that the Ln^3+^ ion had C_1_ local symmetry, which was consistent with the number of crystal-field components of the ^5^D_0_-^7^F_0_, ^5^D_0_-^7^F_1_, and ^5^D_0_-^7^F_2_ transitions (1, 3, and 5, respectively) [44].

**Figure 5:**
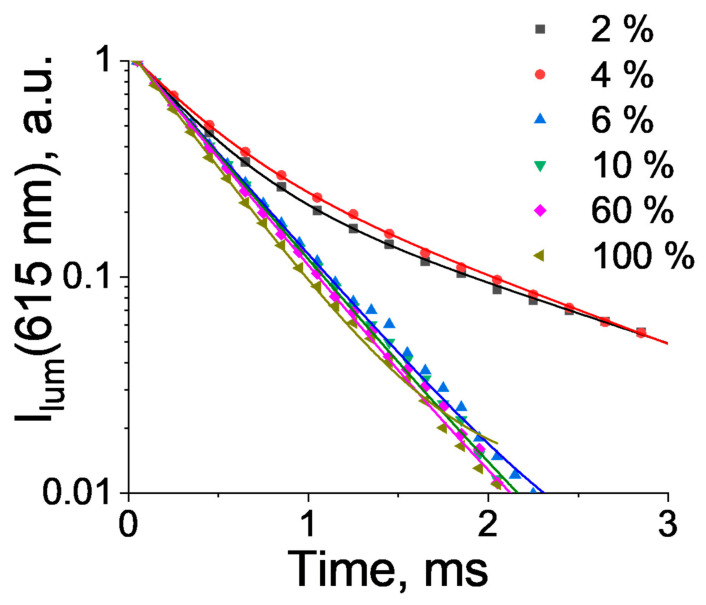
The 615 nm luminescence decay curves of heterometallic europium(III)–lutetium(III) terephthalates at 2, 4, 6, 10, 60, and 100 at % Eu.

The luminescence decay curves of the (Eu_x_Lu_1−x_)_2_bdc_3_·nH_2_O phosphors monitored at 615 nm (^5^D_0_-^7^F_2_ transition) are presented in Figure 5 (λ_ex._ = 280 nm). At a low Eu^3+^ concentration (2 and 4 at %), the decay curves were fitted by a double exponential function (1), whereas the decay curves of the Eu-Lu terephthalates containing 6–100 at % Eu^3+^ were fitted by a single exponential function (2):

(1) $${I=I}_{1}\cdot e^{-\frac{t}{\tau_{1}}}{+I}_{2}\cdot e^{-\frac{t}{\tau_{2}}}$$

(2) $${I=I}_{1}\cdot e^{-\frac{t}{\tau_{1}}}$$

where τ_1_ and τ_2_ are the observed ^5^D_0_ lifetimes (Table 2).

The Eu-Lu terephthalates containing 6–100 at % Eu^3+^ had ^5^D_0_ lifetimes of 0.390–0.459 ms and luminescence quantum yields of 10–16%. The measured PLQY of the Eu_2_bdc_3_·4H_2_O was comparable with the literature data [31,38,45]. The ^5^D_0_ lifetime values and the luminescence quantum yields decreased with an increase in the Eu^3+^ concentration due to the energy migration between the Eu^3+^ ions and subsequent quenching by impurities and defects. We demonstrated in this work that Eu^3+^ ions predominantly existed in the Ln_2_bdc_3_·4H_2_O phase in the europium(III)–lutetium(III) terephthalates containing 6–100 at % Eu^3+^, in which the presence of a single Eu^3+^ coordination site resulted in a single ^5^D_0_ lifetime. Interestingly, the europium(III)–lutetium(III) terephthalates containing 2–4 at % Eu^3+^ were characterized by two ^5^D_0_ lifetimes. One emission decay component (τ_1_ = 0.392–0.367 ms) was close to the value observed for the europium(III)–lutetium(III) terephthalates containing 6–100 at % Eu^3+^, while another component (τ_2_ = 1.602–1.878 ms) was 4–4.8 times larger. In the Eu-Lu terephthalates containing 2–4 at % Eu^3+^, the Eu^3+^ ions were distributed between the Ln_2_bdc_3_·4H_2_O and Ln_2_bdc_3_ crystalline phases. Therefore, τ_1_ and τ_2_ could be assigned to the Eu^3+^ ions located in the Ln_2_bdc_3_·4H_2_O and Ln_2_bdc_3_, respectively. The water molecules in the Ln_2_bdc_3_·4H_2_O structure were coordinated with the Eu^3+^ ion and quenched the Eu^3+^ luminescence due to efficient energy transfer to high-energy O-H stretching vibrational modes of coordinated water molecules [46,47]. In the Ln_2_bdc_3_ crystalline phase, the Eu^3+^ ion was coordinated only with oxygen atoms of carboxylic groups of terephthalate ions. The efficient quenching of Eu^3+^ ions by water molecules in the Ln_2_bdc_3_·4H_2_O structure resulted in a significant decrease in the Eu^3+^ ion ^5^D_0_ lifetime compared to anhydrous Ln_2_bdc_3_.The emission quantum yield of the Eu^3+^ was significantly larger for the Eu-Lu terephthalates doped with a low Eu^3+^ concentration. This observation can be explained by two reasons: the absence of efficient Eu-Eu energy migration and the presence of the Ln_2_bdc_3_ crystalline phase with a significantly smaller nonradiative decay rate compared to the Ln_2_bdc_3_·4H_2_O.

## 3. Materials and Methods

Lutetium (III) chloride hexahydrate and europium (III) chloride hexahydrate were purchased from Chemcraft (Kaliningrad, Russia). Benzene-1,4-dicarboxylic (terephthalic, H_2_bdc) acid (>98%), sodium hydroxide (>99%), nickel(II) chloride hexahydrate (>99%), and EDTA disodium salt (0.1 M aqueous solution) were purchased from Sigma-Aldrich Pty Ltd. (Germany) and used without additional purification. The 0.2M solutions of EuCl_3_ and LuCl_3_ were prepared and standardized using complexometric titration with EDTA. A total of 0.6 mole of sodium hydroxide and 0.3 mole of terephthalic acid were dissolved in distilled water to obtain a 1 L solution of a 0.3 M solution of the disodium terephthalate (Na_2_bdc).

The heterometallic europium(III)–lutetium(III) terephthalates were obtained by mixing 1 mL of 0.2 M EuCl_3_ and LuCl_3_ aqueous solutions taken in stoichiometric ratios with 2 mL of 0.3 M Na_2_bdc water solution (Table 3). White precipitates of heterometallic europium(III)–lutetium(III) terephthalates were separated from the reaction mixture using centrifugation (4000× *g*) and washed using deionized water 5 times. All samples were driedat 60 °C.

The Eu^3+^/Lu^3+^ ratios in the heterometallic europium(III)–lutetium(III) terephthalates were confirmed using energy-dispersive X-ray spectroscopy (EDX) (EDX spectrometer EDX-800P, Shimadzu, Japan) (Table 4). The Eu/Lu ratios measured via EDX were consistent with the ratios of Eu^3+^/Lu^3+^ taken for the synthesis in the form of EuCl_3_ and LuCl_3_ aqueous solutions (Table 3). The X-ray powder diffraction (XRD) measurements were performed on a D2 Phaser (Bruker, USA) X-ray diffractometer using Cu K_α_ radiation (λ = 1.54056 Å). The thermogravimetry curves were obtained using a TG 209 F1 Libra thermo-microbalance (Netzsch, Germany). The luminescence spectra were recorded with a Fluoromax-4 fluorescence spectrometer (Horiba Jobin Yvon, Japan). Lifetime measurements were performed with the same spectrometer using a pulsed Xe lamp (pulse duration: 3 µs). The absolute values of the photoluminescence quantum yields were recorded using a Fluorolog 3 Quanta-phi device. All measurements were performed at 25 °C.

## 4. Conclusions

In this work, we reported on the photoluminescence properties of luminescent antenna MOF—heterometallic europium(III)–lutetium(III) terephthalates. The series of (Eu_x_Lu_1−x_)_2_bdc_3_·nH_2_O (x = 0–1) was synthesized in the aqueous solution. At Eu^3+^ concentrations of 1–40 at %, the heterometallic europium(III)–lutetium(III) terephthalates were formed as a mixture of the (Eu_x_Lu_1−x_)_2_bdc_3_ and (Eu_x_Lu_1−x_)_2_bdc_3_·4H_2_O crystalline phases. At higher Eu^3+^ concentrations, a single crystalline phase was formed: (Eu_x_Lu_1−x_)_2_bdc_3_·4H_2_O. All the synthesized samples containing Eu^3+^ demonstrated a bright red emission corresponding to the ^5^D_0_-^7^F_J_ (J = 0–4) transitions of Eu^3+^ ions upon 280 nm excitation into the singlet electronic excited state of terephthalate ions. An analysis of the fine structure of the emission spectra allowed us to conclude that the Eu^3+^ ions were unevenly distributed between the Ln_2_bdc_3_ and Ln_2_bdc_3_·4H_2_O phases: the Ln_2_bdc_3_·4H_2_O crystalline phase was enriched by Eu^3+^ ions. The local symmetry of the Eu^3+^ ions in the heterometallic Eu-Lu terephthalates was proposed based on a careful analysis of the fine structure of the emission spectra and the structural data. We demonstrated that the ^5^D_0_ excited state lifetimes were 4–4.8 times larger for Eu^3+^ in the Ln_2_bdc_3_ crystalline phase than in Ln_2_bdc_3_·4H_2_O due to the absence of luminescence quenching of the Eu^3+^ by coordinated water molecules. The luminescence quantum yields of terephthalate ions decreased with an increase in the europium concentration from 2 to 100 at % Eu^3+^ (λ_ex._ = 280 nm).

## Figures and Tables

**Table 1:** Unit cell parameters with calculation errors for (Eu_x_Lu_1−x_)_2_bdc_3_·nH_2_O refined for Eu_2_bdc_3_·4H_2_O crystalline phase.

χ_Eu_ (%)	a, Å	b, Å	c, Å	α	β	γ	V, Å^3^
100	6.1904 ±0.0019	9.856 ±0.003	10.251 ±0.003	101.673 ±0.027	90.273 ±0.028	104.796 ±0.025	591.13 ±0.22
90	6.1862 ±0.0019	9.845 ±0.003	10.236 ±0.003	101.583 ±0.027	90.300 ±0.028	104.727 ±0.025	589.63 ±0.22
60	6.1727 ±0.0018	9.815 ±0.003	10.206 ±0.003	101.553 ±0.027	90.418 ±0.028	104.657 ±0.025	585.00 ±0.22
40	6.1635 ±0.0018	9.783 ±0.003	10.179 ±0.003	101.497 ±0.027	90.472 ±0.028	104.651 ±0.025	580.75 ±0.22
20	6.1405 ±0.0018	9.738 ±0.003	10.145 ±0.003	101.570 ±0.027	90.562 ±0.028	104.617 ±0.025	573.90 ±0.21
10	6.1411 ±0.0018	9.7178 ±0.003	10.1334 ±0.003	101.626 ±0.026	90.461 ±0.028	104.590 ±0.025	572.13 ±0.21
6	6.1313 ±0.0018	9.714 ±0.003	10.130 ±0.003	101.582 ±0.026	90.474 ±0.027	104.608 ±0.025	570.784 ±0.21

**Table 2:** The lifetimes of excitation state 5D0 of Eu^3+^ in heterometallic europium(III)–lutetium(III) terephthalates at 2, 4, 6, 10, 60, and 100 at % Eu.

χ_Eu_ (%)	τ_1_, ms	τ_2_, ms	PLQY, %
100	0.390		10 ± 1
60	0.435		11 ± 1
10	0.449		12 ± 1
6	0.459		16 ± 1
4	0.392	1.602	22 ± 1
2	0.367	1.878	22 ± 1

**Table 3:** The heterometallic europium(III)–lutetium(III) terephthalates’ synthesis conditions.

χ_Eu_, %	V(0.2M EuCl_3_), mL	V(0.2M LuCl_3_), mL
0	0	1.00
1	0.01	0.99
2	0.02	0.98
3	0.03	0.97
4	0.04	0.96
5	0.05	0.95
6	0.06	0.94
7	0.07	0.93
8	0.08	0.92
9	0.09	0.91
10	0.10	0.90
20	0.20	0.80
30	0.30	0.70
40	0.40	0.60
50	0.50	0.50
60	0.60	0.40
70	0.70	0.30
80	0.80	0.20
90	0.90	0.10
100	1.00	0

**Table 4:** Eu^3+^ atomic fraction (relative to the total amount of Eu^3+^ and Lu^3+^) in heterometallic europium(III)–lutetium(III) terephthalates taken during synthesis and obtained from EDX data.

χ_Eu_ (%), Taken	χ_Eu_ (%), EDX
0	0
2	2.07 ± 0.21
4	3.9 ± 0.4
5	4.7 ± 0.5
6	6.3 ± 0.6
10	10.2 ± 1.0
20	19.3 ± 1.9
40	37 ± 4
80	80 ± 8
100	100

## Data Availability

The data presented in this study are available in the article.

## Sample Availability

Samples of the reported compounds are available from the authors.
